# Supplementary material for: Nanofibrous cosmetic face mask for transdermal delivery of nano gold: synthesis, characterization, release and zebra fish employed toxicity studies
Source: R Soc Open Sci. 2020 Sep 30;7(9):201266. doi: 10.1098/rsos.201266 (PMC7540761; doi:10.1098/rsos.201266)
Supplement: Nano Gold release studies [file rsos201266supp1.docx]

Nanofibrous cosmetic face mask for transdermal delivery of nano gold: Green synthesis, characterization, release and Zebra fish embryo employed toxicity studies

D.C. Manatunga^a^, V. U. Godakanda^a^, H.M.L.P.B Herath^a^, Rohini M. de Silva^a^, Chen-Yu Yeh^b^, Jiann-Yeu Chen^c^, A.A. Akshitha De Silva^b^, S. Rajapaksha^d^, Renuka Nilmini^d^, K. M. Nalin de Silva^a*^

1. *Centre for Advanced Materials and Devices, Department of Chemistry, University of Colombo, Colombo 00300, Sri Lanka.*
2. *Department of Chemistry, National Chung Hsing University, Taichung 402, Taiwan.*
3. *Research Centre for Sustainable Energy and Nanotechnology (RCSEN), National Chung Hsing University, Taichung 402, Taiwan.*
4. *Department of Engineering Technology, Faculty of Technology, University of Sri Jayawardenepura, Sri Lanka*

Results


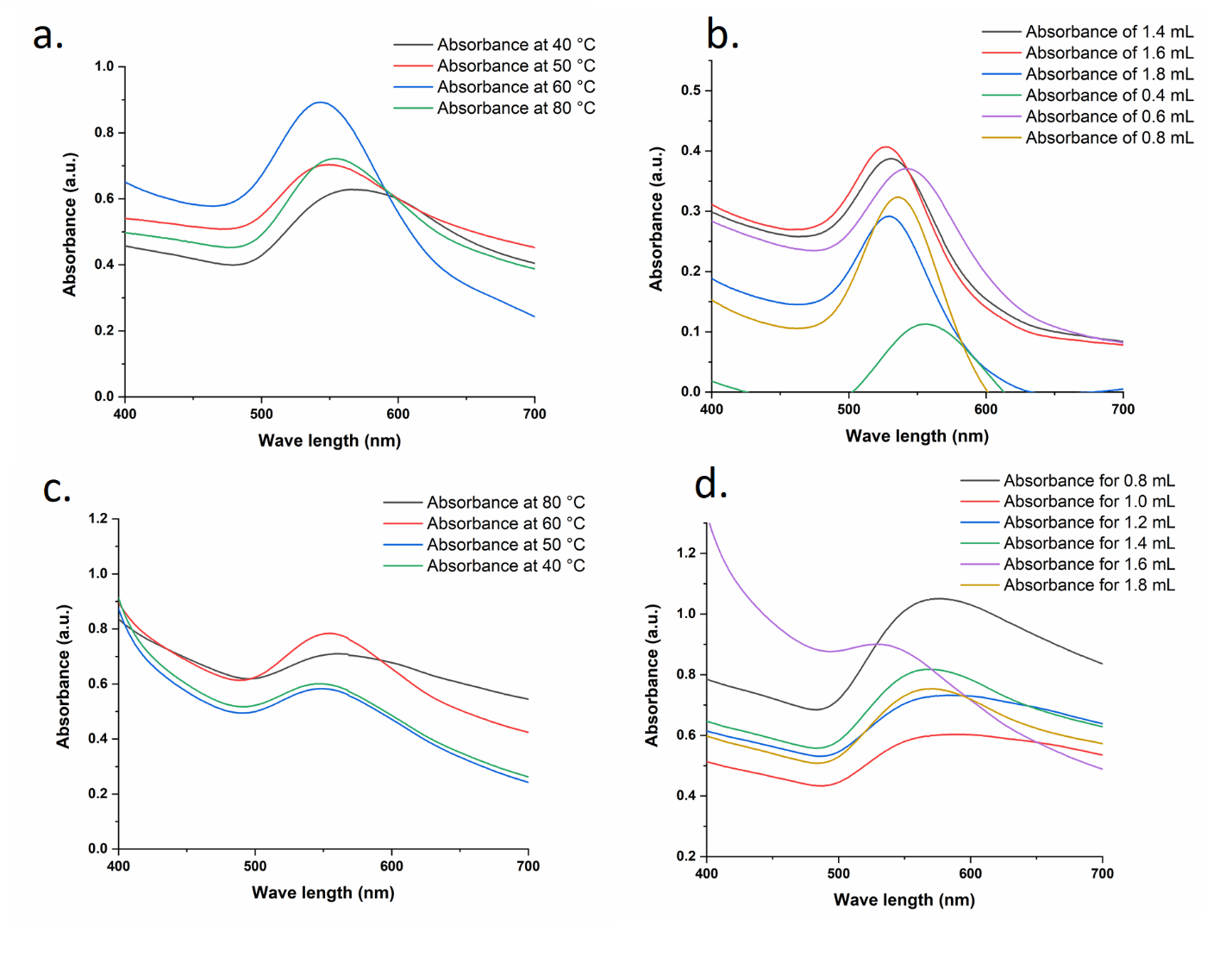


**Figure S1**. UV visible absorption spectra of Au NPs synthesized using green tea extract and orange peel extract a) temperature optimization in the range of 40 °C – 80 °C and b) volume optimization at 60 °C (optimum temperature) of GtAu NPs and c) temperature optimization in the range of 40 °C – 80 °C and b) volume optimization at 40 °C (optimum temperature) of OpAu NPs

**Table S1**

Details of spinning solutions used in this work with the resulted viscosity and conductivity. All solutions were prepared in DDW

| Sample | Amount of PEO | Amount of gelatin | Amount of AA | Amount of COL | Amount of Au | Viscosity  (cP) | Conductivity  (mS/cm) |
| --- | --- | --- | --- | --- | --- | --- | --- |
| PEO | 4.5 wt % | - | - | - | - | 5700 | 0.094 |
| PEO-Gel | 4.0 wt% | 0.5 wt% | - | - | - | 6100 | 0.122 |
| PEO-Gel-AA-COL | 7.3 wt% | 0.5 wt% | 1.0% | 0.2% | - | 2300 | 0.125 |
| PEO-Gel-GtAu NPs | 7.3 wt% | 0.5 wt% | 1.0% | 0.2% | 0.75 mg | 4800 | 0.589 |
| PEO-Gel- OpAu NPs | 7.3 wt% | 0.5 wt% | 1.0% | 0.2% | 0.75 mg | 5050 | 0.497 |
| PEO-Gel-AA-COL-GtAu NPs | 7.3 wt% | 0.5 wt% | 1.0% | 0.2% | 0.50 mg | 2240 | 0.567 |
| PEO-Gel-AA-COL-GtAu NPs | 7.3 wt% | 0.5 wt% | 1.0% | 0.2% | 0.75 mg | 2256 | 0.577 |
| PEO-Gel-AA-COL-GtAu NPs | 7.3 wt% | 0.5 wt% | 1.0% | 0.2% | 1.0 mg | 2231 | 0.581 |
| PEO-Gel-AA-COL-OpAu NPs | 7.3 wt% | 0.5 wt% | 1.0% | 0.2% | 0.5 mg | 2209 | 0.551 |
| PEO-Gel-AA-COL-OpAu NPs | 7.3 wt% | 0.5 wt% | 1.0% | 0.2% | 0.75 mg | 2300 | 0.587 |
| PEO-Gel-AA-COL-OpAu NPs | 7.3 wt% | 0.5 wt% | 1.0% | 0.2% | 1.0 mg | 2134 | 0.630 |

**Table S2**

Electrospinning optimum conditions resulted for each polymer solution

| System | Distance (cm) | Voltage (kV) | Flow rate (mL/hr) |
| --- | --- | --- | --- |
| PEO | 18 | 11.9 | 0.3 |
| PEO-Gel | 18 | 18 | 0.3 |
| PEO-Gel-AA-COL | 18 | 19.2 | 0.3 |
| PEO-Gel-GtAu NPs | 15 | 18.8 | 0.3 |
| PEO-Gel-OpAu NPs | 13 | 15.9 | 0.3 |
| PEO-Gel-AA-COL-GtAu NPs (0.5 mg Au) | 13 | 15.9 | 0.3 |
| PEO-Gel-AA-COL-GtAu NPs (0.75 mg Au) | 13 | 15.9 | 0.3 |
| PEO-Gel-AA-COL-GtAu NPs (1.0 mg Au) | 13 | 15.9 | 0.3 |
| PEO-Gel-AA-COL-OpAu NPs (0.5 mg Au) | 13 | 13.5 | 0.3 |
| PEO-Gel-AA-COL-OpAu NPs (0.75 mg Au) | 13 | 13.8 | 0.3 |
| PEO-Gel-AA-COL-OpAu NPs (i.0 mg Au) | 13 | 13.8 | 0.3 |


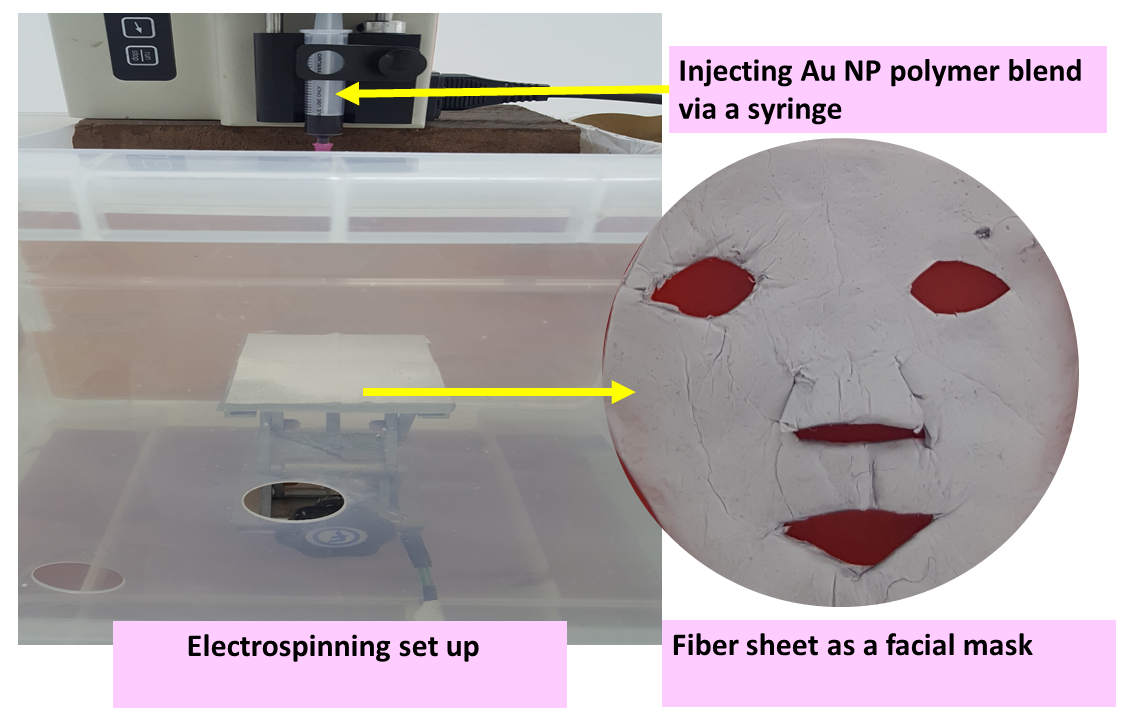


Figure S2. Electrospinning set up used for the preparation of Au nanofiber masks and the resulted thin fiber mat which could be used as a facial mask


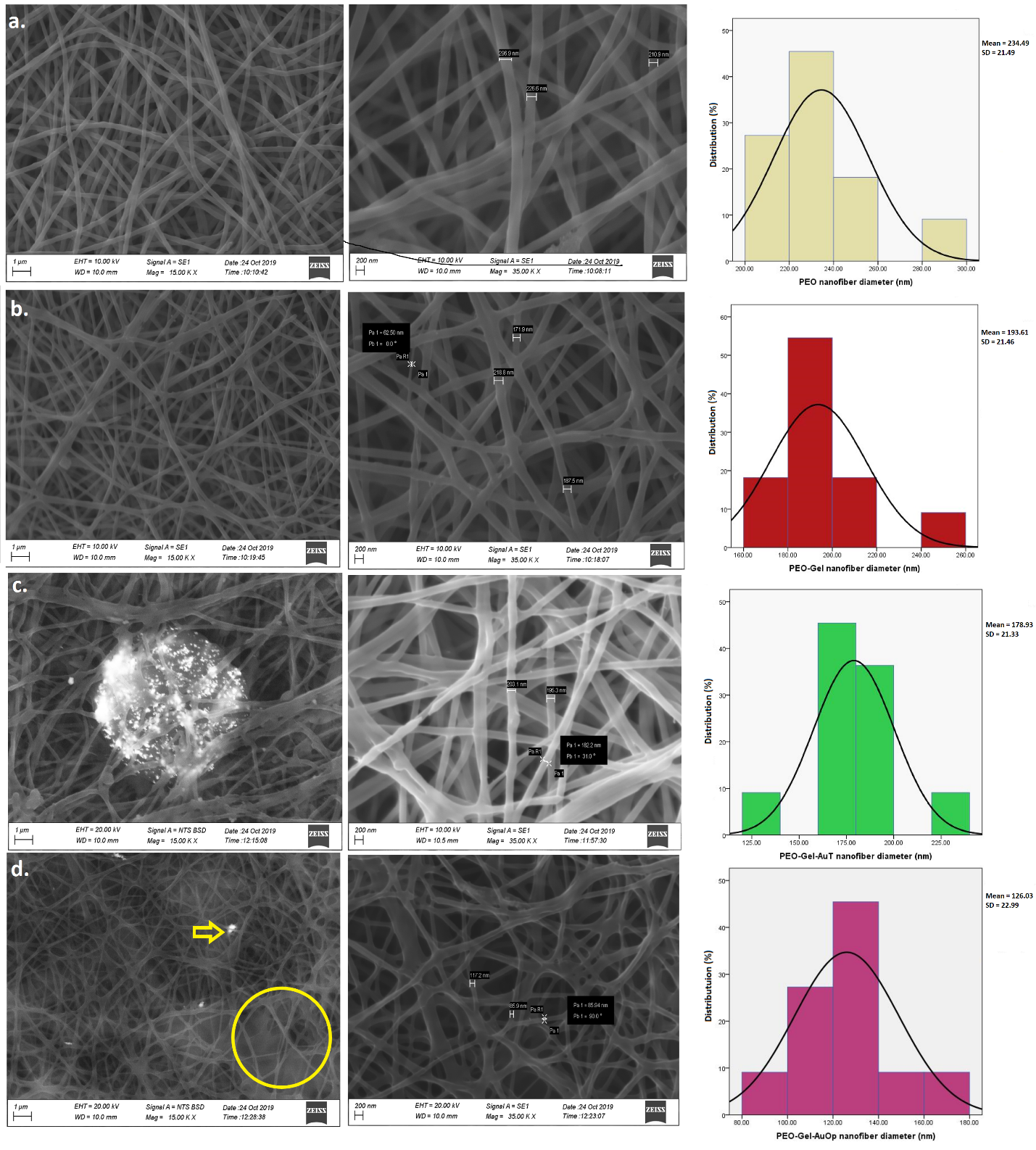
 Figure S3- SEM images and size distribution histograms of nanofiber systems a) PEO system, b) PEO-Gel, c) PEO-Gel-GtAu NPs system, d) PEO-Gel-OpAu NPs system (arrow indicates the entrapment of Au NPs inside the nanofibers, whereas circled area indicates the merging of nanofibers with unspun polymer clumps)


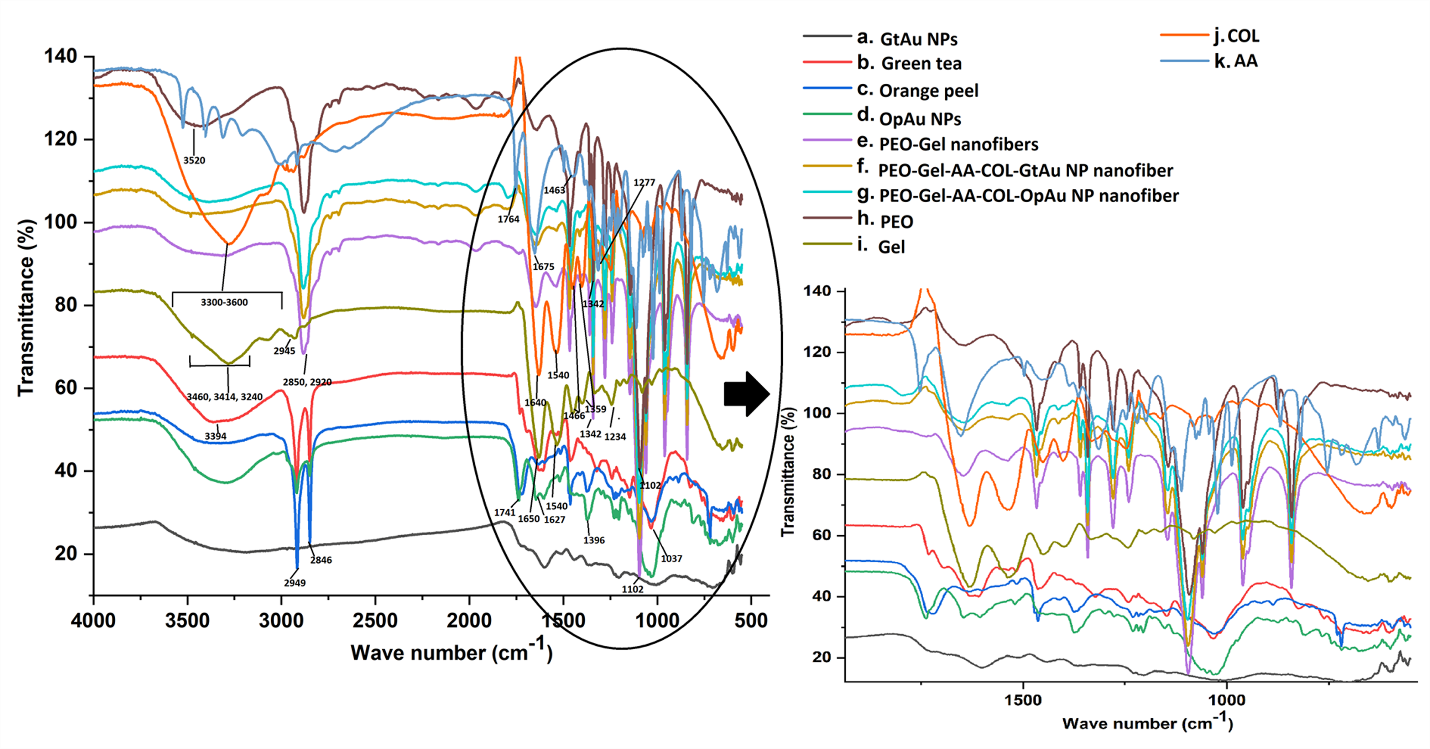


Figure S4. FT-IR spectra of neat polymers and polymer blended Au NPs incorporated nanofiber systems


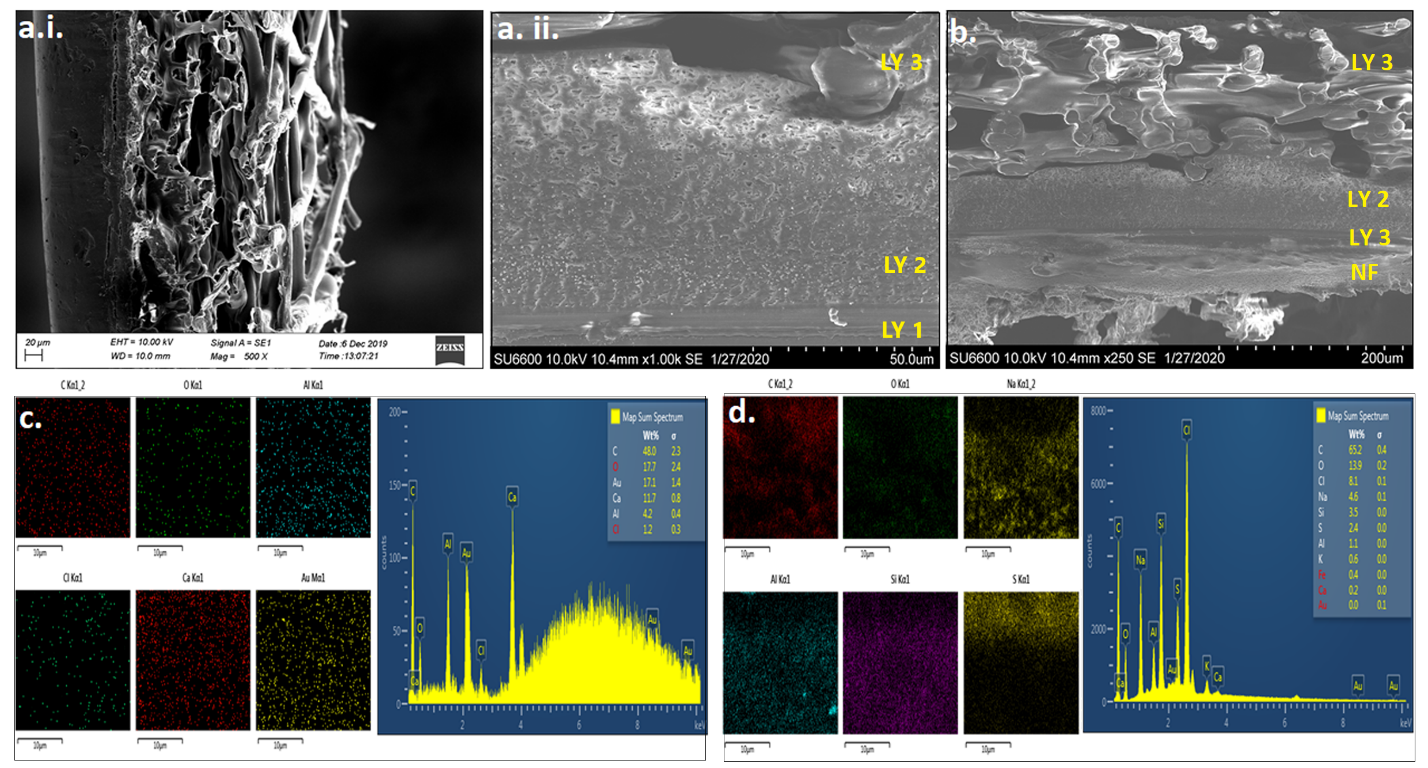


Figure S5- Analysis of Au NP diffusion through the Strat membrane a. i. and a. ii. cross sectional analysis of Strat membrane indicating the three layers (LY(layer)-LY1, LY2 and LY3), b. cross sectional analysis of nanofiber on top of the Strat membrane (NF-Nanofiber), c. elemental analysis of Strat membrane kept in contact with PEO-Gel-AA-COL-GtAu nanofiber and d. elemental analysis of Strat membrane kept in contact with PEO-Gel-AA-COL-OpAu nanofiber





Figure S6. Radical scavenging activity of PEO-Gel-AA-COL-GtAu nanofiber system, PEO-Gel-AA-COL-OpAu nanofiber system and Ascorbic acid standard expressed as a function of percentage inhibition activity Vs the different concentration of the compounds of interest. Results are expressed as mean ± SD of three separate experiments.
